# Silent Carriers: The Role of Rodents in the Emergence of Zoonotic Bacterial Threats

**DOI:** 10.3390/pathogens14090928

**Published:** 2025-09-15

**Authors:** Shereen Basiouni, Alfonso J. Rodriguez-Morales, Awad A. Shehata, Phelipe Magalhães Duarte

**Affiliations:** 1 Center for Preclinical Research, German Cancer Research Center, 69120 Heidelberg, Germany; 2 Faculty of Health Sciences, Universidad Científica del Sur, Lima 15307, Peru; arodriguezmo@cientifica.edu.pe; 3 Grupo de Investigación Biomedicina, Faculty of Medicine, Fundación Universitaria Autónoma de las Américas-Institución Universitaria Visión de las Américas, Pereira 660003, Colombia; 4 TUM School of Natural Sciences, Bavarian NMR Center (BNMRZ), Structural Membrane Biochemistry, Technical University of Munich, 85748 Garching, Germany; dr_awadali_1@yahoo.com; 5 Postgraduate Program in Animal Bioscience, Federal Rural University of Pernambuco (UFRPE), Recife 52171-900, Pernambuco, Brazil

**Keywords:** rodents, one health, climate change, zoonoses, bacteria

## Abstract

Rodents are recognized as significant reservoirs for a broad range of zoonotic pathogens, including bacteria, viruses, and parasites, many of which have substantial implications for human and animal health. The intensifying interaction between humans and rodent populations, fuelled by urbanization, climate change, and global trade, has amplified the risk of zoonotic disease transmission. This review compiles and examines current knowledge on key rodent-borne bacterial diseases, including leptospirosis, rat-bite fever, plague, salmonellosis, tularemia, Lyme disease, rickettsioses, Babesiosis, and associated parasitic infections such as toxoplasmosis and Chagas disease. Each disease is analyzed in terms of its etiology, transmission, clinical manifestations, diagnostic tools, and treatment options, with a particular focus on the impact of environmental changes. Emphasizing a One Health perspective, this work highlights the importance of interdisciplinary approaches to the surveillance, prevention, and control of rodent-borne zoonoses, particularly in the context of increasing climate variability and anthropogenic pressures.

## 1. Introduction

Rodents (order: Rodentia) are among the most prolific and adaptable mammalian species, thriving in nearly all environments, including densely populated urban areas. Their ubiquitous presence and close association with human settlements make them critical contributors to the ecology of zoonotic diseases. As reservoirs and vectors of a wide range of pathogens, including bacteria, viruses, and parasites, rodents pose a persistent and often underestimated threat to global health [1].

Rodent-borne bacterial infections, such as leptospirosis, rat-bite fever, plague, salmonellosis, tularemia, and Lyme disease, have re-emerged in recent years, driven by climatic fluctuations, ecological disruptions, and increased human encroachment into natural habitats [2]. These diseases vary widely in clinical presentation, ranging from mild flu-like symptoms to severe systemic conditions with high mortality rates. Given their dual role as ecological reservoirs and disease vectors, understanding the biology, behaviour, and epidemiological significance of rodents is vital for public health and biodiversity management [3].

Rodents, the most significant order of mammals, comprising over 2200 recognized species, account for approximately 42% of global mammalian diversity [4]. These mammals have a heterogeneous and cosmopolitan distribution, with their expansion closely linked to increasing human interaction. Beyond synanthropic species, other rodents inhabit wild, urban, and rural environments, further increasing contact with animals and humans [5,6]. These animals serve as natural reservoirs of infectious diseases, transmitting pathogens both directly and indirectly, as well as through vectors [3,7].

This review examines the epidemiology, transmission dynamics, diagnosis, and control of rodent-borne pathogens, providing a comprehensive synthesis of current knowledge on this topic. Special attention is given to the impact of climate change on pathogen prevalence and distribution, emphasizing the urgency of adopting integrated One Health strategies. Recognizing the hidden threat posed by rodent-borne zoonoses is essential for mitigating their impact and safeguarding both human and animal populations.

## 2. Leptospirosis

### 2.1. Etiology

Leptospirosis is a zoonotic bacterial disease with a worldwide distribution, affecting approximately 1 million people and causing around 60,000 deaths annually [8]. The highest estimates of disease morbidity and mortality were observed in the Global Burden of Disease (GBD) regions of South and Southeast Asia, Oceania, the Caribbean, Andean, Central, and Tropical Latin America, as well as East Sub-Saharan Africa [8].

Morphologically, leptospires are thin, flexible, Gram-negative, motile bacteria, with one or both ends shaped like a hook, giving them a characteristic question-mark appearance [9]. The 64 known species of *Leptospira* are grouped into two pathogenic subclades [P1 (Pathogenic species) and P2 (Intermediate pathogens)] and two saprophytic subclades (S1 and S2). Most *Leptospira* species responsible for infecting humans and animals result from infections by virulent P1 species, such as *Leptospira interrogans*, *Leptospira kirschneri*, *Leptospira borgpetersenii*, and *Leptospira noguchii* [10,11,12].

### 2.2. Epidemiology

Rodents are most frequently infected by *Leptospira interrogans* serovar Icterohaemorrhagiae. However, other species, such as *L. borgpetersenii*, *L. kirschneri*, *L. broomii*, and *L*. *santarosai*, have also been identified in wild Norway rats [13,14,15,16]. The first three species are also commonly found in other wild rodent populations [16]. Rats, along with other rodents, serve as the primary reservoir for *Leptospira*; however, not all species of *Leptospira* are present in rats [17].

*Leptospira* lives in the renal tubules of these animals, which act as reservoirs, playing a fundamental role in the epidemiology of the disease by contaminating environments through the excretion of *Leptospira* in their urine. This bacterium can survive in aquatic environments and soil for up to two months. Leptospires are typically transmitted to humans through direct contact with the urine of infected rodents, which usually remain asymptomatic, or indirectly via urine-contaminated water or soil [18]. The bacteria enter the human body through minor skin abrasions or mucous membranes of the eyes, mouth, or nose. Cases of transmission from pet rats to humans in close contact with them have been documented [19,20]. The highest prevalence rates remain in tropical, developing countries where leptospirosis cases are on the rise [21,22].

### 2.3. Clinical Signs

**Figure 1:**
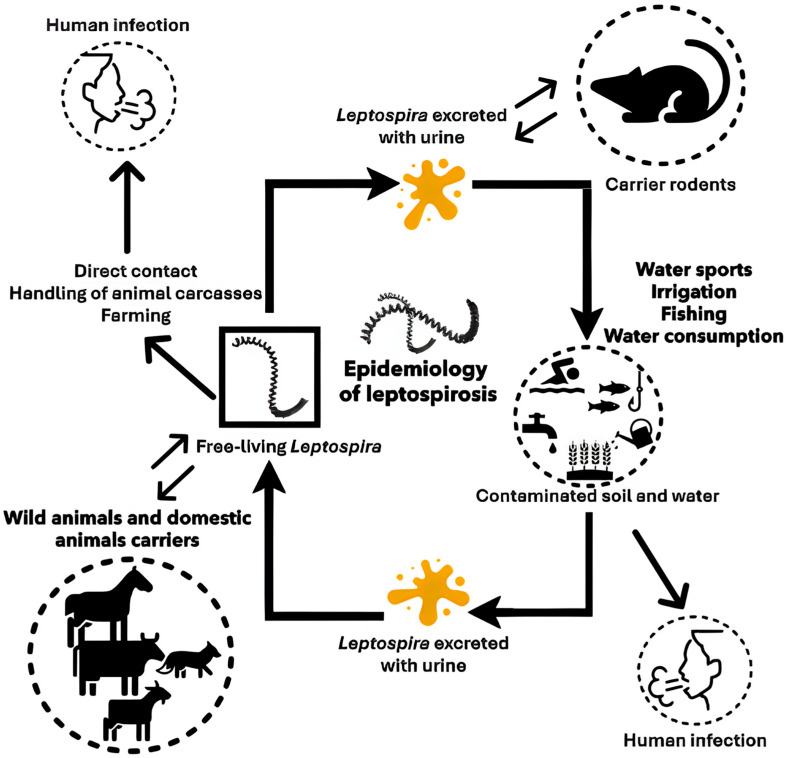
Transmission of *Leptospira* to humans and the role of the rodents. Rodents, along with wild and domestic animals, serve as primary reservoirs of *Leptospira*, harboring the bacteria in their renal tubules and excreting them in urine. Human infection occurs through direct contact with infected urine or indirectly via contaminated water or soil, with bacteria entering through skin abrasions or mucous membranes.

The incubation period for leptospirosis typically ranges from 7 to 14 days, though it can vary between 2 and 30 days. The majority (approximately 90%) of human infections are either asymptomatic or present with mild flu-like symptoms. However, severe forms can occur, potentially leading to life-threatening complications such as pulmonary hemorrhage, liver and kidney failure (Weil’s disease), as well as meningitis, pancreatitis, or encephalitis [18]. Transplacental transmission may also occur, resulting in abortion or stillbirth [18] (Figure 1). In animals, leptospirosis can cause a range of clinical manifestations and pathological changes. In calves, infection may result in hemolytic anemia and hemoglobinuria, with interstitial nephritis as a sequela, and late-term abortion in cows (combined for clarity). In dairy cows, mastitis syndrome and occasional abortions may occur. In pigs, the most significant losses arise from abortions, stillbirths, neonatal mortality, and interstitial nephritis (reworded for conciseness) [23]. In dogs, most infections are subclinical; however, symptomatic cases are characterized by lethargy, fever, anorexia, and polyuria/polydipsia, which may progress to multiple organ dysfunction, including acute kidney injury, cholestatic liver dysfunction, pancreatitis, varying degrees of pulmonary hemorrhage, myositis, and occasionally uveitis [24]. In horses, the disease may manifest as febrile illness, reproductive losses, and neonatal disease [24,25,26].

### 2.4. Diagnosis

Diagnosis of leptospirosis can be made through direct pathogen detection, such as polymerase chain reaction (PCR)-based identification of pathogenic *Leptospira* DNA from blood, urine, cerebrospinal fluid, or tissue samples (e.g., kidney). Direct detection can also be performed through bacterial culture using the Ellinghausen-McCullough-Johnson-Harris (EMJH) medium, though due to the slow growth rate of *Leptospira*, this method is not suitable for rapid diagnosis [27]. In contrast, PCR testing provides faster results and allows for pathogen typing. Leptospires can also be detected through dark-field microscopy in blood or urine samples; however, this method has limited sensitivity and specificity. Alternative methods include silver staining techniques such as Steiner, Levaditi, Dieterle, and Warthin-Starry staining, as well as immunohistological examinations. Indirect detection relies on serological methods to identify antibodies against *Leptospira*. The microscopic agglutination test (MAT) is the gold standard for serological diagnosis, while enzyme-linked immunosorbent assays (ELISA) are also commonly used.

### 2.5. Treatment, Prevention, and Control

Symptomatic leptospirosis is treated with antibiotics as early as possible, as the bacteria generally respond well to antimicrobial therapy. Effective antibiotics include tetracyclines, β-lactams, and macrolides, particularly azalides [28].

Leptospirosis is a disease closely linked to the concept of One Health, so environmental balance is fundamental to understanding the disease dynamics. In this sense, reports indicate that climate change will lead to drastic changes in global ecosystems with unpredictable and incalculable impacts. Based on the disease dynamics, the intensification of events related to rainfall and floods is a critical point for the increase in leptospirosis cases [29]. Therefore, to achieve significant results in controlling leptospirosis, it must be addressed within the context of One Health.

Commercially available vaccines are composed of whole inactivated *Leptospira* and are approved for human use only in a few countries, such as Japan, Cuba, France, and China [30]. However, these vaccines induce only serovar-specific protection and short-term immunity, requiring annual immunization. Furthermore, serious side effects are reported after administration, limiting their use [31].

### 2.6. Recommendations

Given the complex epidemiology and environmental persistence of leptospirosis, a comprehensive, integrated approach (replaced “comprehensive and integrated” → “comprehensive, integrated” for conciseness) is essential for effective prevention and control. The author recommends implementing One Health strategies that acknowledge the interconnectedness of human, animal, and environmental health. The author recommends implementing One Health strategies that acknowledge the interconnectedness of human, animal, and environmental health. This includes controlling rodent populations, especially in urban and peri-urban areas, by reducing access to food sources, sealing entry points, and effectively managing waste and sanitation. Public education campaigns are crucial for raising awareness about risk factors, such as exposure to contaminated water or soil, and for promoting protective behaviors, like wearing boots and gloves in flood-prone or high-risk areas.

In endemic regions, early diagnosis using rapid molecular techniques, such as PCR, should be prioritized to improve clinical outcomes. Timely antibiotic treatment remains a key tool in managing infections. Where available, vaccines may be considered for high-risk groups; however, their limitations, including serovar-specific protection and potential side effects, must be taken into account. Finally, given the strong link between climate events (e.g., heavy rainfall and flooding) and outbreaks, climate adaptation strategies should be integrated into public health planning to anticipate and mitigate future spikes in leptospirosis incidence.

## 3. Rat-Bite Fever

### 3.1. Etiology

Rat-bite fever (RBF) is a systemic infectious disease primarily caused by two bacterial species: *Streptobacillus moniliformis* and *Spirillum minus* [32]. However, past case reports describing *Spirillum* minus are based solely on morphological observations made with dark-field microscopy, lacking molecular or phenotypic confirmation. *S. moniliformis* is now recognized as the primary causative agent of RBF, with wild rats serving as its primary reservoir [33,34]. Other *Streptobacillus* species of interest include *S. notomytis*, which can also cause RBF but is primarily transmitted by the black rat (*Rattus rattus*), and *S. felis*, which leads to a similar illness but originates from cats [35,36].

Rats harbor *S. moniliformis* in their oral cavity and throat, typically without exhibiting symptoms. The bacteria spread easily among rats through social interactions, although stressed or injured rats may develop abscesses or other bacterial infections [37]. House mice (*Mus musculus*) can contract *S. moniliformis* only through contact with infected rats, but they do not play a significant role in the epidemiology of human RBF. Susceptibility among mouse strains varies, with some developing fever, arthritis, abscesses, and septicemia upon infection [38].

### 3.2. Epidemiology

The prevalence of *S. moniliformis* in wild rat populations varies significantly, with reported rates ranging from 2% to 92%, depending on the location and the methods used in the studies [33,39]. Variations in detection techniques, such as antibody tests, genomic analysis, or bacterial cultures, as well as the specific rat populations studied (urban vs. rural, wild vs. pet store or feeder rats), contribute to these discrepancies. Although serological testing can be highly sensitive, no commercial diagnostic tests are currently available. Furthermore, the incidence of infection and the minimum infectious dose for humans remain unknown.

In Germany, it is estimated that between 30,000 and 50,000 bite wounds are treated each year, with bites from dogs and cats being the most common. Less than 10% of these injuries are caused by rodents such as rats, hamsters, and rabbits. Notably, 59% of the affected individuals are children or adolescents [40]. The overall risk of infection following a rat bite is estimated to be between 10% and 20%, with 30% to 60% of cases resulting in polymicrobial infections, which include both aerobic and anaerobic bacteria [40]. Pet stores and feeder rats are not routinely screened for *S. moniliformis*, meaning the bacterium is likely present in pet rats and can be transmitted to humans, primarily through close contact.

**Figure 2:**
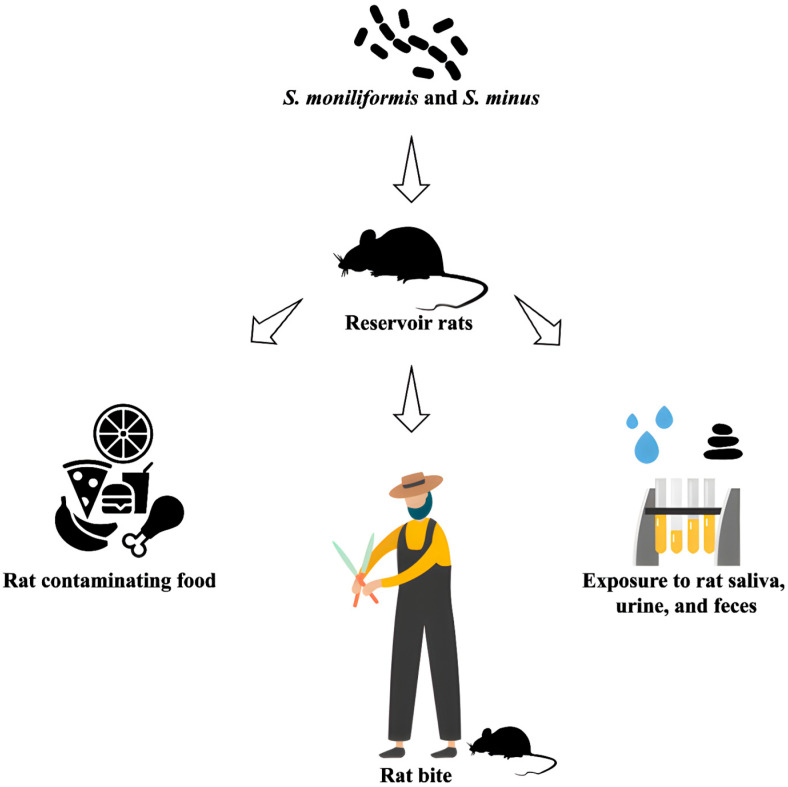
Transmission of Rat-Bite Fever (RBF): Overview of how *Streptobacillus moniliformis* and *S. minus* are transmitted from rats to humans through bites, scratches, or contact with contaminated materials.

High-risk groups include veterinarians, animal caretakers, sewer workers, farmers, and homeless individuals. Additionally, due to the increasing popularity of pet rats, children are at an elevated risk [41,42]. Infections can also occur through indirect exposure to contaminated saliva, urine, feces, food, or materials from cages [43]. The bacteria can enter the body through direct contact, skin wounds, or mucous membranes, such as those found in the eyes, nose, or mouth [43] (Figure 2). Diagnostic samples can be collected from various sources, including blood, mucosal swabs, fine-needle aspirates, wound secretions, abscess fluid, synovial fluid, or cerebrospinal fluid [43]. Blood cultures should be taken repeatedly, as initial bacterial growth may not always be detected successfully [43].

### 3.3. Clinical Aspects

The incubation period for *S. moniliformis* infection spans from 3 to 21 days [44,45]. Common symptoms include recurrent fever, migratory polyarthralgia (affecting approximately 50% of cases), and a rash on the hands and feet (observed in about 75% of cases) [44,45]. These key symptoms are often accompanied by general complaints, such as headaches, muscle pain, and elevated inflammatory markers, including neutrophilia, increased erythrocyte sedimentation rate, and high levels of C-reactive protein [31,32]. If left untreated, severe complications can arise, including abscesses, hepatitis, nephritis, pneumonia, meningitis, encephalitis, osteomyelitis, bacteremia, spondylodiscitis, and inflammation of the heart [44,45].

A foodborne variant known as Haverhill fever (*Erythema arthriticum epidemicum*) results from consuming contaminated food or water. This variant presents additional gastrointestinal symptoms, including pharyngitis, vomiting, and diarrhea. The estimated mortality rate for this condition is around 10%, although severe cases can have significantly higher fatality rates [46].

In laboratory mice infected with *S. moniliformis*, clinical signs ranging from septic lymphadenitis to polyarthritis and multiorgan microabscesses can be observed, leading to septicemia, cachexia, and death [47]. In nonhuman primates, rat-bite fever caused by *S. moniliformis* has been reported in a rhesus monkey (*Macaca mullata*) with valvular endocarditis and a titi monkey (*Callicebus* sp.) with septic arthritis [48].

### 3.4. Diagnosis

The PCR technique can be used to detect *S. moniliformis* in skin lesions [32]. Furthermore, the identification of *S. moniliformis* bacteria can be performed through culture from plasma or joint fluid. To do this, the sample must be injected into a culture medium free of sodium polyanethole sulfonate, an anticoagulant used in culture media because it inhibits the growth of the organism [49]. Other tests, such as gas–liquid chromatography and 16S rRNA sequencing, are more sensitive than culture [50,51]. *S. minus* cannot be cultured, and the only methods for identifying it are dark-ground microscopy from blood smears, lesions, or lymph nodes, and Giemsa staining [51].

### 3.5. Treatment, Prevention, and Control

When diagnosed promptly, RBF can typically be treated without complications. The preferred antibiotics for this condition include penicillin, doxycycline, or ceftriaxone, which can be administered either orally or intravenously [52,53]. However, decolonizing infected rodents poses challenges, as these treatments do not guarantee long-term eradication of the bacteria [54]. Despite being treatable, Rat-Bite Fever (RBF) is often referred to as a “diagnostic dilemma” because its symptoms are nonspecific and resemble those of the flu. Additionally, there is a lack of systematic case reporting in both human and veterinary medicine [55]. The bacterium responsible for RBF grows slowly and requires microaerophilic conditions, along with specialized culture media, which complicates laboratory diagnosis. Moreover, routine molecular and serological diagnostic tools are not widely available [34].

### 3.6. Recommendations

To prevent Rat-Bite Fever, the author recommends raising awareness, particularly among high-risk groups, including veterinarians, pet owners, and individuals exposed to rodents. Individuals should avoid direct contact with rats’ saliva, urine, or feces and use protective gloves when handling rodents or cleaning cages. Children should be supervised around pet rats to prevent bites or close face contact. Pet stores and breeders should screen rats for *Streptobacillus moniliformis* to minimize the risk of transmission. In cases of rodent bites or symptoms such as fever and rash, clinicians should consider RBF and utilize appropriate diagnostic tools, including PCR or specialized cultures. Improved case reporting and access to reliable diagnostics are also essential for better monitoring and control.

## 4. *Yersinia pestis*

Human plague, caused by the bacterium *Yersinia pestis*, has resulted in three pandemics in history, including the first plague of Justinian (around 541 AD), the second Black Death (around 1347 AD), and the third plague (around 1880 AD) [56]. While often associated with historical events, plague is currently considered a re-emerging disease, with increasing human infection rates, particularly in Africa [57].

### 4.1. Etiology

*Y. pestis* is a Gram-negative bacterium belonging to the Enterobacteriaceae family, non-spore-forming, immobile coccobacillus cultured in broth, with bipolar staining using Giemsa or Wayson, with an optimal growth temperature between 26–28 °C, and an optimal pH of approximately 7.5 [58].

Indeed, *Y. pestis* is the most notorious species within the *Yersinia* genus, which includes two other species pathogenic to humans: *Yersinia enterocolitica* and *Yersinia pseudotuberculosis* [59]. *Y. enterocolitica* is an enteropathogenic bacterium primarily affecting the gastrointestinal tract. It is commonly found in soil environments and infects various mammalian and avian species, with an exceptionally high prevalence in domestic pig populations [60]. In humans, large outbreaks of *Y. enterocolitica* infections are typically associated with the consumption of contaminated food [61]. *Y. pseudotuberculosis*, like *Y. enterocolitica*, is also a gastrointestinal pathogen. It is widely distributed in the environment, particularly in soil and water, where it can persist for extended periods [62]. This bacterium has developed several adaptation mechanisms that enable it to survive under biotic and abiotic conditions in the soil, which differ significantly from those encountered during its host-associated life cycle [62]. *Y. pseudotuberculosis* primarily causes gastrointestinal infections following the ingestion of contaminated food.

### 4.2. Epidemiology

**Figure 3:**
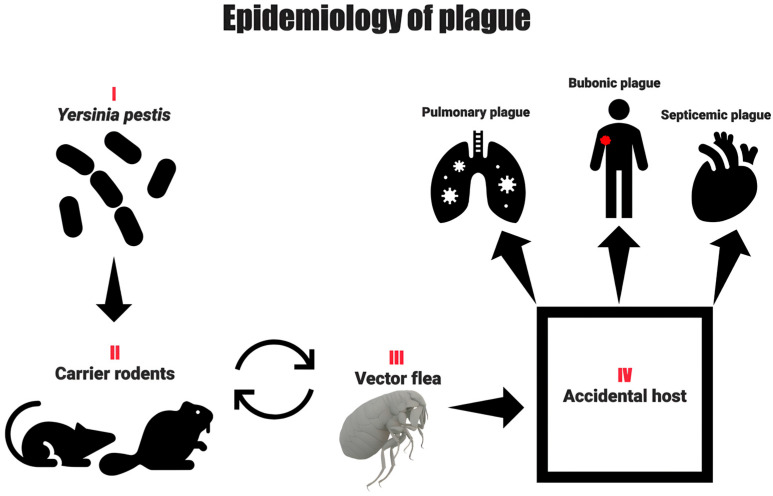
Transmission routes of plague: Visual representation of plague transmission from infected rodents to humans primarily via flea bites, with possible secondary transmission through contact with infected animals or inhalation of infectious droplets in pneumonic plague cases.

Plague has a global distribution, with the three most endemic countries being Madagascar, Congo, Uganda, Peru, Tanzania, and the United States [63]. Between 2010 and 2019, the World Health Organization received 4547 cases with a 17% fatality rate. Madagascar experienced four major outbreaks of primary pneumonic plague, affecting nearly 2000 people and causing 137 deaths, including one outbreak involving a streptomycin-resistant *Yersinia pestis* strain. The region with the highest concentration of diversity is China, which hosts a wide variety of hosts, including rodents [64,65,66]. Mammals are the most common host species, with about 351 species capable of acting as reservoirs. Among these species, 279 are rodents [66,67]. Additionally, the bacterium is maintained in flea populations that become infected by feeding on animals already infected with the bacterium [48,50,51]. Besides fleas, other blood-sucking arthropods, such as *Argasidae*, *Gamasidae*, *Ixodidae*, *Anoplura*, and *Heteroptera*, can harbor the bacterium, but they are not significant in epizootics and disease outbreaks [65] (Figure 3).

By the end of 2021, there were 12 types of natural plague foci in the mainland of China, located in 322 county-level divisions of 19 provincial-level administrative divisions (PLADs), covering a total of 1,587,666.67 square kilometers. Between 2002 and 2021, plague epizootics or positive indications were identified for 12 types of natural plague foci in 196 county-level divisions of 16 PLADs [68]. An average of seven human plague cases are reported each year in the United States. Most plague cases occur in the western part of the country, mainly in northern New Mexico and Arizona [69]. 

The plague can be transmitted from a patient with pneumonic plague to other individuals through droplet transmission [70,71,72]. In addition to aerosol transmission, contact between susceptible individuals and infected tissue, whether of animal or human origin, poses a high risk of infection to humans. Contagion by direct contact could cause a contained spread to occur among relatives and close communities of people who take care of their dead [73]. In the environment, approximately 80 species of fleas can be infected with the bacterium [74], which is considered the most efficient method of transmission [75].

In the next century, the combination of climate change and anthropogenic activities is expected to result in a significant increase in pathogen spillover and the emergence of zoonotic diseases [76,77]. Following this line, plague cases are closely linked to climatic anomalies and the richness of rodent species [78]. Climate change can result in unfavorable situations, such as food shortages and famine, which can trigger migratory waves and, consequently, increased interaction between plague vectors [79,80]. Therefore, due to the acceleration of climate change, it is essential to maintain constant surveillance of *Y. pestis*, its hosts, and at-risk populations.

### 4.3. Clinical Signs

In 1940, G. P. Rudnev [81] proposed an epidemiological classification of plague that remains relevant to public health practice. He divided plague into three main categories based on the mode and extent of dissemination: (A) predominantly local forms, including cutaneous, bubonic, and cutaneous-bubonic types, which are typically peripheral and rarely spread externally; (B) internally disseminated or generalized forms, comprising primary and secondary septicemic plague; and (C) externally disseminated forms, which are often highly contagious and include primary pneumonic, secondary pneumonic, and intestinal plague. While this classification supports practical applications in disease control, subsequent research has noted that “pure” cutaneous and intestinal forms have not been observed in clinical practice [82].

i. Septicemic Plague: Clinically, the septicemic form of plague, caused by *Y. pestis*, resembles septicemia caused by other Gram-negative bacteria. Affected individuals may present with hyperemia, chills, headache, apathy, and gastrointestinal disturbances. There is some evidence that patients with septicemic plague have a higher incidence of abdominal pain than patients with bubonic plague [83].
ii. Pneumonic Plague: The pneumonic form has an incubation period of 1 to 3 days. Primary pneumonic plague is a rare but highly lethal form of the disease, mainly transmitted through droplets and aerosols via close contact (within 2 to 5 feet) with infected individuals. This form of the disease progresses rapidly from a febrile illness to severe pneumonia, producing cough and bloody sputum [84].
iii. Bubonic Plague: Bubonic plague is the most frequent clinical form, occurring 2 to 10 days after inoculation with *Y. pestis* [85]. Clinical signs include hyperemia, myalgias, arthralgias, and apathy [74]. Another clinical sign is lymphadenomegaly, also known as “buboes,” with the femoral (~31%) and inguinal (~24%) lymph nodes being the most frequently affected, followed by the axillary (~22%) and cervical (~9%) lymph nodes [74,86].

In dogs, clinical signs may include fever, lethargy, anorexia, lymphadenopathy, vomiting, diarrhea, and abscesses [87]. Felids, including domestic cats, are highly susceptible, presenting with lymphadenopathy and unexplained high fever [88].

### 4.4. Diagnosis

The diagnosis of the disease requires laboratory confirmation, with the best method being a sample of pus from a bubo, blood, or sputum. Among the diagnostic methods, the rapid diagnostic test (RDT), which aims to detect the F1 antigen of *Y. Pestis*, is a practical tool that can be widely used, ensuring a wide range of tests and diagnostic coverage for the population, and can provide results in approximately 15 min [89].

The use of molecular tests, developed to ensure greater accuracy of results, such as conventional PCR, aims to amplify the genes pla, caf1, inv, and yopM [90,91], can ensure greater sensitivity in diagnoses, in addition to real-time PCR, which can be performed in just 2 h [92]. Other molecular tools, such as loop-mediated isothermal amplification (LAMP) technology, have also been developed [93].

The standard test for *Y. pestis* is bacterial isolation. *Y. pestis* grows in usual culture media; however, the use of selective agar supplemented with cefsulodin–irgasan–novobiocin (CIN) favors the isolation of the bacterium in polymicrobial samples, such as sputum. According to WHO recommendations, the patient should rinse their mouth with water before collecting the sample to reduce contamination by oral flora. After 2 or 3 days of incubation at 28 °C, suspected colonies on CIN agar can be identified by biochemical tests, PCR, and specific phage lysis for *Y. pestis* [94].

### 4.5. Treatment, Prevention, and Control

The recommended treatment includes the use of streptomycin or gentamicin in adult patients, including those who are immunocompromised and pregnant women. These drugs can be administered to children at a reduced dose. Alternatively, the combination of doxycycline, ciprofloxacin, and chloramphenicol can also be used for adults and children [95].

Preventive measures include informing people when zoonotic plague is present in their environment and advising them to take precautions against flea bites and avoid handling animal carcasses. Generally, people should be advised to avoid direct contact with infected body fluids and tissues. When handling potentially infected patients and collecting specimens, standard precautions should be applied. Surveillance and control require investigating the animal and flea species implicated in the plague cycle in the region and developing environmental management programs to understand the natural zoonotic cycle of the disease and limit its spread. Active long-term surveillance of animal foci, coupled with a rapid response during animal outbreaks, has successfully reduced the number of human plague outbreaks.

To effectively and efficiently manage plague outbreaks, it is crucial to have an informed and vigilant health care workforce (and community) to quickly diagnose and manage patients with infection, to identify risk factors, to conduct ongoing surveillance, to control vectors and hosts, to confirm diagnosis with laboratory tests, and to communicate findings with appropriate authorities [57].

Plague vaccines have been in existence for over a century. First-generation vaccines, while potentially effective, are limited by high rates of reactogenicity [96]. To date, there are no prequalified plague vaccines. The vaccine candidates in the most advanced stages are adjuvanted subunit vaccines based on the F1 and LcrV proteins [97,98,99,100]. All current vaccine candidates lack efficacy data in humans.

### 4.6. Recommendations

In areas with confirmed or suspected cases of zoonotic plague, it is essential to raise public awareness about the presence of the disease in the environment. People should avoid handling animal carcasses and any contact with potentially contaminated tissues or body fluids. When in or near forests or tall vegetation, individuals should minimize exposure by avoiding direct contact with the vegetation and wearing long-sleeved, light-colored clothing that fully covers the feet, ankles, and legs. The use of insect repellents containing DEET (N,N-diethyl-meta-toluamide) and thymol is recommended, as well as the use of citrus-scented essential oils around the home to help deter fleas. Pet care is also crucial—dogs should be taken to the vet regularly, and all pets should be kept up to date on deworming treatments. To reduce the rodent population, avoid accumulating garbage, especially materials like cardboard and old magazines. Lastly, avoid contact with wild rodents to further reduce the risk of exposure to the plague.

## 5. *Salmonella*

### 5.1. Etiology

Approximately 1 million people worldwide contract salmonellosis each year, resulting in 60,000 deaths [101]. Although the infection is commonly acquired through the ingestion of contaminated food, many cases are acquired through contact with animals [102,103]. It is a bacterium belonging to the Enterobacteriaceae family, rod-shaped, Gram-negative, facultative anaerobe, non-spore-forming, with an optimal growth temperature between 35 and 37 °C [104,105,106].

*Salmonella* can be divided into typhoidal and non-typhoidal serovars (NTS). Within the typhoidal serovar, we have *Salmonella Typhi* and *Salmonella Paratyphi* A, which cause typhoid and paratyphoid fever, respectively [107]. As for the non-typhoidal serovars of *Salmonella*, we have *Typhimurium* and *Enteritidis* [108]. The most prevalent species is *S. enterica*, with about 1500 serotypes, responsible for 99% of human and animal infections [109,110].

The genus *Salmonella* is divided into two species, *S. enterica* and *S. bongori*, with *S. enterica* being the most clinically relevant. It is further classified into six subspecies and over 2600 serotypes based on the Kauffmann–White scheme. Human-adapted typhoidal serovars include *S. Typhi* and *S. Paratyphi* A, B, and C, which are typically restricted to humans and cause enteric (typhoid) fever. In contrast, non-typhoidal *Salmonella* (NTS) serovars, such as *S. Typhimurium* and *S. Enteritidis*, have broad host ranges and are commonly acquired from animals or animal-derived food products. Other important NTS include *S. Heidelberg*, *S. Newport*, *S. Dublin*, *S. Infantis*, *S. Agona*, and *S. Javiana*, among others, which are also capable of zoonotic transmission. Rodents play a crucial role in maintaining and disseminating many non-typhoidal serovars across farms and food production systems, underscoring their importance as reservoirs [107,108,109,111].

### 5.2. Epidemiology

The main reservoir of *Salmonella* is warm-blooded animals, such as rodents [112]. On farms, rodents such as house mice are a significant source of infection. Among these species, the house mouse (*Mus musculus*) was identified as one of the rodents responsible for transmitting *Salmonella* Enteritidis infections among farm animals [113]. Other species, such as the roof rat (*Rattus rattus*), are also sources of *S. Enteritidis* infections [114,115]. The species *Rattus rattus*, *Rattus norvegicus*, and *Mus musculus domesticus* have been identified as sources of several *Salmonella* serotypes on poultry and swine farms [115,116,117,118].

The Typhimurium serotype is frequently isolated from captive and wild rodents, with intermittent fecal excretion lasting from weeks to months [119,120,121,122,123,124]. Pet rodents are an underrecognized source of human *Salmonella* infection [125]. In a *Salmonella Typhimurium* outbreak in Canada, rodents used to feed snakes supplied by a network of rodent breeders in Ontario were identified as the source of the outbreak. The cases likely acquired their illness through direct or indirect contact with these rodents [126]. In another study, *Salmonella Typhimurium* of the *S. enterica* serotype was isolated from 28 patients, 13 of whom reported exposure to hamsters, mice, or pet rats [125]. In Thailand, rats captured in eight traditional wet markets had a *Salmonella* prevalence of 49.10%, with 30% of these being *Salmonella Typhimurium* (30%) and 12.7% *S. Weltevreden* [127]. Of the 299 *Salmonella* isolates from rodents in several UK studies, *S. Enteritidis* and *S. Typhimurium* accounted for 58.5% and 28.4%, respectively [124].

**Figure 4:**
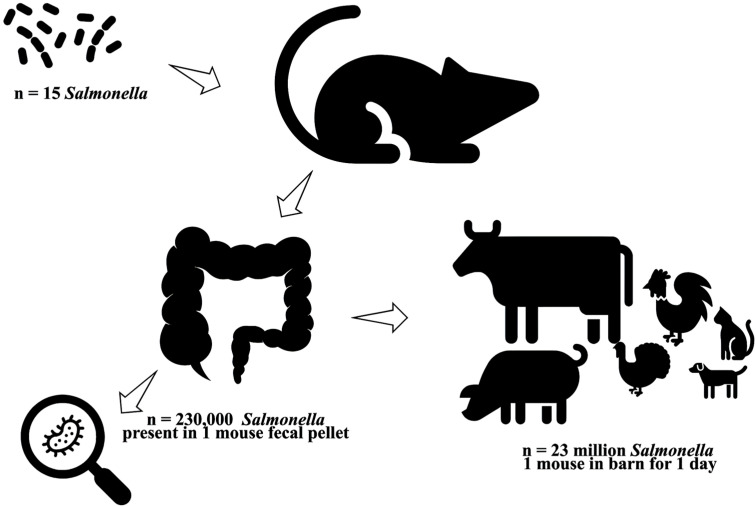
Rodents as reservoirs, amplifiers, and vectors of *Salmonella* Infections. Rodents can significantly amplify environmental contamination: as few as 15 *Salmonella* cells can infect a rodent, and a single fecal pellet may contain approximately 230,000 bacteria. Given that a rodent may excrete up to 100 pellets per day, it can release over 23 million *Salmonella* cells into the environment within 24 h, heavily contaminating barns and other settings and increasing the risk of food- or waterborne outbreaks.

Rodents can significantly amplify *Salmonella* contamination in the environment. As few as 15 *Salmonella* cells are sufficient to infect a rodent. Once colonized, a single fecal pellet from the infected animal can contain around 230,000 bacteria. Given that a rodent may excrete up to 100 pellets in a day, it can release over 23 million *Salmonellae* into its surroundings within 24 h. This substantial shedding can heavily contaminate barns and other environments, increasing the risk of food- or waterborne disease outbreaks (Figure 4).

*Salmonella* is widely distributed in domestic and wild animals. They are prevalent in food animals, such as poultry, pigs, and cattle, as well as in pets, including cats, dogs, birds, and reptiles like turtles. *Salmonella* can circulate through the entire food chain from animal feed and primary production to households, food-service establishments, and institutions. In humans, *Salmonella* infections generally occur through the consumption of contaminated foods of animal origin (primarily eggs, meat, poultry, and milk). However, salmonellosis can also occur through the consumption of other foods, including green vegetables contaminated with manure. Person-to-person transmission can also occur through the faecal–oral route, which is of great epidemiological importance. Humans can also develop salmonellosis when individuals come into contact with infected animals, including pets. These infected animals often do not show signs of disease [128]. 

Like other zoonotic diseases, the increase in salmonellosis is related to rising temperatures [128,129]. Studies have shown that a 1 °C increase in temperature increases the estimated risk of salmonellosis by between 3% and 13% [130]. Climate change plays a significant role in the increase in *Salmonella* cases, leading to serious consequences for public health [131,132].

### 5.3. Clinical Signs

In humans, salmonellosis can cause symptoms such as fever, nausea, vomiting, abdominal pain, and headache [112]. Clinical signs typically begin 12 to 36 h after ingesting *Salmonella*, with an average illness duration of 2 to 7 days. Symptoms are mild, and the disease generally has a self-limiting course in affected individuals, who recover without specific treatment for salmonellosis [128]. However, in some cases, particularly in children and elderly patients, aggravating factors such as dehydration can be life-threatening. Although large *Salmonella* outbreaks usually attract media attention, the disease remains underreported, and the actual number of cases is unknown [128].

Non-typhoidal *Salmonella* (NTS) infections in humans are most frequently associated with self-limiting gastroenteritis, characterized by diarrhea, abdominal cramps, fever, and vomiting. However, in vulnerable populations such as young children, the elderly, and immunocompromised individuals, NTS can lead to invasive disease with bacteremia, focal infections (e.g., meningitis, osteomyelitis), and, rarely, septicemia [133,134]. Typhoidal *Salmonella* serovars, including *S. Typhi* and *S. Paratyphi* A, B, and C, cause enteric fever, a systemic illness characterized by prolonged fever, malaise, abdominal pain, hepatosplenomegaly, and, in severe cases, intestinal perforation. These invasive presentations underscore the importance of appropriate antimicrobial therapy in high-risk patients and those with enteric fever or bacteremia [133,135,136,137].

In cattle, *Salmonella* infections can result in clinical signs of enterocolitis, septicemia, and abortion [138]. Pneumonia is a relatively common clinical manifestation in calves [139,140]. Horses may present fever, diarrhea, or leukopenia [141]. Most *Salmonella* infections in dogs and cats are asymptomatic. In these animals, the most common clinical signs are acute enterocolitis and septicemia, with consequent endotoxemia. Rare syndromes include conjunctivitis in cats and in utero infections in dogs and cats, resulting in abortions, stillbirths, or the birth of weak kittens. Acute enterocolitis usually develops within 3 to 5 days of exposure and is limited to mucosal invasion. It manifests as watery or mucoid diarrhea, containing blood in severe cases, accompanied by vomiting, fever (40–41 °C), loss of appetite or anorexia, lethargy, abdominal pain, and progressive dehydration [142].

### 5.4. Diagnosis

One of the diagnostic methods used for *Salmonella* is microbial culture. Most diagnostic laboratories use a combination of selective enrichment broth followed by subculture to one or more selective agar plates, and then identify presumptive *Salmonella* colonies using biochemical techniques [143]. The use of molecular biology techniques, such as PCR, enables the detection of *Salmonella* in a wide range of samples, including water and human stool samples [144,145,146]. It is recommended that PCR after overnight enrichment in a nonselective broth be adopted as the gold standard, and that all positive PCR samples be cultured using selective enrichment [147,148].

### 5.5. Treatment, Prevention, and Control

In the setting of NTS bacteremia or disseminated disease, initial therapy should be with third-generation cephalosporins, such as ceftriaxone, for at least 7 to 10 days. Once bacterial susceptibilities are known, antibiotic treatment can be transitioned to azithromycin or a fluoroquinolone.

For enteric fever, the antibiotic treatment of choice is a fluoroquinolone. In cases of resistance, alternative antibiotics, such as third-generation cephalosporins and azithromycin, are recommended as an alternative. Fluoroquinolones are not used as frequently in children as they are in adults, and alternatives such as azithromycin are often preferred. The typical treatment duration is 10 to 14 days. In cases of severe enteric fever with symptoms of delirium, obtundation, stupor, or shock, additional treatment with corticosteroids may be considered. Dexamethasone at a dose of 3 mg/kg, followed by 1 mg/kg every 6 h for 48 h, has been shown to reduce mortality [149].

Prevention requires control measures at all stages of the food chain, from agricultural production to processing, manufacturing, and preparation of foods in both commercial establishments and at home. Preventive measures for *Salmonella* in the home are similar to those used against other foodborne bacterial diseases (see recommendations for food handlers below). The contact between infants/young children and pet animals that may be carrying *Salmonella* needs careful supervision [128].

Wild rodents can act as hosts for a variety of pathogens and transmit them to other farm animals through their feces, which can contaminate food and water throughout the farm. Therefore, frequent disinfection is required [150]. In addition, it is necessary to adopt measures such as the disposal of garbage and bedding, adequately filling any holes or openings to prevent access by mice, and storing supplies in a clean area to prevent access by rodents [151].

Only licensed vaccines are available against *S. Typhi*, the leading cause of typhoid fever. These include the orally administered live attenuated Ty21a vaccine, which protects against *S. Typhi* and offers some cross-protection against *S. Paratyphi* B, but not against *S. Paratyphi* A [152,153]. Additionally, there are injectable Vi capsular polysaccharide and conjugate vaccines, which are highly effective in protecting against *S. Typhi*. The Typbar-TCV vaccine, composed of *S. Typhi* Vi polysaccharide conjugated to tetanus toxoid, was over 80% effective in Phase 3 trials in children aged 9 months to 16 years [154].

### 5.6. Recommendations

To prevent rodent infestation and related health risks, it is crucial to eliminate factors that encourage their presence and reproduction. This includes maintaining clean and organized environments, avoiding the accumulation of garbage and debris, storing food in airtight containers, and sealing any cracks, holes, or other access points. Garbage should be collected regularly at the regional level, and food must be stored safely, with no leftovers left exposed. Areas that are not frequently visited should be cleaned routinely. Additionally, practicing proper hygiene is essential: wash hands thoroughly, especially before handling food and after using the bathroom, and ensure kitchen utensils are cleaned adequately before food preparation. Safe food and water practices must also be followed. Only drink filtered or boiled water. Avoid consuming raw or undercooked meat. Ensure eggs are well-cooked, and drink only pasteurized or boiled milk.

## 6. *Francisella*

*Tularemia* is a zoonosis bacteriosis caused by the bacterium *Francisella tularensis*. The disease occurs naturally in lagomorphs (rabbits and hares) and rodents, especially microtine rodents such as voles, vole rats, and muskrats, and also in beavers. Furthermore, several other mammals can be infected by the bacterium, and it has been isolated from birds, fish, amphibians, arthropods, and protozoa [155].

### 6.1. Etiology

Tularemia is a zoonotic bacterial disease caused by *Francisella tularensis*, a highly infectious, Gram-negative coccobacillus found throughout the Northern Hemisphere [156].

The bacterium is classified into two types: Type A (subsp. tularensis), associated with a terrestrial transmission cycle and found throughout North America, and Type B (Subsp. *holarctica*), associated with aquatic environments and found in North America, Australia, Japan, and Europe [157,158,159]. *Francisella tularensis* Subsp. *Tularemia* (Type A) is primarily associated with lagomorphs in North America and is typically transmitted by ticks, biting flies, or direct contact with infected animals. It is highly virulent for humans and domestic rabbits, and most isolates ferment glycerol. *Francisella tularensis* subsp. *holarctica* (Type B) occurs mainly in aquatic rodents (beavers, muskrats) and voles in North America, and lagomorphs (hares) and rodents in Eurasia. It is primarily transmitted through direct contact or by arthropods (primarily ticks and mosquitoes), but may also be transmitted via inhalation, contaminated water, or contaminated food. It is less virulent for humans and domestic rabbits, and does not ferment glycerol [155]. The species and subspecies of the genus *Francisella* are organized in the table below (https://lpsn.dsmz.de/search?word=Francisella), (accessed on 22 April 2025).

### 6.2. Epidemiology

Rodents and lagomorphs are maintenance hosts of *F. tularensis*, with small wild mammals being important reservoirs [160,161]. Due to their high reproduction rates and short lifespan, rodents are ideal hosts for *F. tularensis* [162]. In Finland, specific DNA of *F. tularensis* was detected in field voles (*Microtus agrestis*) [163]. The most common reservoirs are *Arvicola terrestris* (water vole) and *Microtus arvalis* (field vole), as well as *Rattus rattus* (black rat) in Europe, *Arvicola terrestris*, *Mus musculus*, and hares in the *Lepus* genus in Russia, and hares in the *Sylvilagus* genus in North America [164].

**Figure 5:**
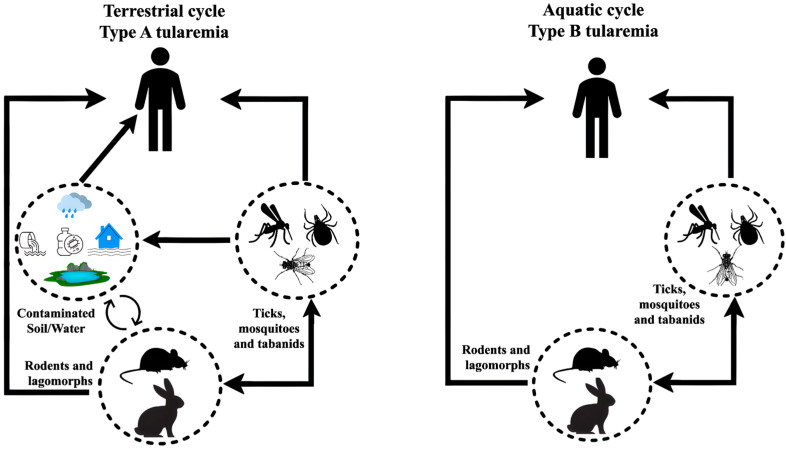
Transmission of tularemia to humans by ticks and the life cycle of *F. tularensis* in nature. Two primary transmission cycles exist: the terrestrial cycle, involving wild rodents, lagomorphs, and arthropod vectors, and the aquatic cycle, in which contaminated water sources play a crucial role in human infections.

Rabbits, hares, and rodents are especially susceptible and often die in large numbers during outbreaks [154]. Transmission can occur through direct contact with infected animals, via ticks, mosquitoes, and fleas, or by ingesting contaminated water. People can become infected in several different ways, including bites from ticks and deer flies, as well as contact with infected animals (especially rodents, rabbits, and hares) [154]. In regions with Type B *Tularemia*, ingesting water from lakes and rivers, as well as consuming contaminated vegetables without proper hygiene, poses a significant health risk [165,166]. Additionally, transmission can occur by inhaling contaminated aerosols and through contact with the skin of infected animals (Figure 5).

It is essential to strengthen scientific and research capacity on climate change and health to provide a more accurate assessment of the impact of climate change on vector- and rodent-borne infectious diseases, particularly in the context of One Health [167].

### 6.3. Clinical Signs

In sensitive animals, clinical signs of severe depression can be observed, followed by a fatal septicaemia. The disease lasts from 2–10 days, and affected animals die within a few days. Most domestic species do not usually manifest signs of tularemia infection, but they do develop specific antibodies against the bacteria.

Outbreaks with high mortality caused by the Type A organism have occurred in sheep. Among domestic pets, *F. tularensis* infection can result in clinical illness in cats, but it is less commonly seen in dogs. Both have been implicated in the transmission of the disease to humans, from cats to humans, most commonly via bites or scratches, and from dogs via close facial contact, ticks, and the retrieval of carcasses, as well as bites [155].

The first human case of tularemia was confirmed in 1914 in a patient presenting with ocular inflammation, characterized by ulcers and swelling of the eyelid, which progressed to enlarged lymph nodes, abscesses, and hyperemia [168].

In humans, clinical manifestations are divided into forms, including ulceroglandular, glandular, oculoglandular, oropharyngeal, respiratory, and typhoidal [157]. Ulceroglandular tularemia, the most common form of the disease, occurs after the introduction of bacteria into the skin through an arthropod bite or during the handling of infected carcasses, with a low mortality rate [169].

A prevalent form is oropharyngeal tularemia, resulting from the ingestion of contaminated water [156,166]. The respiratory form, resulting from the inhalation of aerosolized bacteria, can reach a mortality rate of up to 30% [157]. The oculoglandular form is rare and typically occurs through the manual transmission of bacteria to the eye or via contaminated droplets. Individuals affected by this form develop painful conjunctivitis and swelling around the eye, associated with edema of the lymph nodes located near the ear or neck [170]. Patients with glandular forms of tularemia do not have an identified ulcer. Approximately 30% of patients with adenopathy progress to lymph node abscesses that require surgical removal [171]. Patients presenting the typhoid or septicemic form may develop hyperemia, prostration, and, sometimes, neurological and/or digestive disorders, such as vomiting, diarrhea, and abdominal pain, without a detected entry point [170].

In dogs, clinical signs such as lethargy, pyrexia, anorexia, and lymphadenopathy may occur [172]. In young adult dogs with experimentally induced infection, the disease manifests similarly to that resulting from natural exposure, but puppies may be affected more severely [173]. In cats, the disease is associated with nonspecific clinical signs such as lethargy, lymphadenopathy, oral ulcers, pyrexia, vomiting, hepatomegaly, and icterus [174]. The localised form manifests with chronically draining subcutaneous abscesses [175].

### 6.4. Diagnosis

Diagnostic methods include serology, culture, and PCR from clinical samples [170,176,177]. Blood cultures can be used in patients with *F. tularensis* bacteremia or from other clinical specimens, such as conjunctival or pharyngeal exudates, lymph node biopsies or suppurations, sputum samples, and cerebrospinal fluid. PCR-based methods are helpful in localized forms of tularemia when exudates or tissue samples can be obtained. Due to the limitations of diagnosis by culture and PCR, serology is the diagnostic method of choice [170,176,177].

### 6.5. Treatment, Prevention, and Control

The treatment of choice for tularemia includes aminoglycosides, tetracyclines, and fluoroquinolones. However, this treatment was primarily developed to address emergencies in the context of bioterrorism. Intravenous (IV) gentamicin is recommended for 7 to 14 days, depending on the severity of the disease, as the most effective treatment [178,179,180,181,182].

To prevent tularemia, it is essential to minimize exposure to ticks, deer flies, and potentially infected animals. When spending time outdoors, such as hiking, camping, or working in nature, individuals should use insect repellents containing DEET, picaridin, IR3535, Oil of Lemon Eucalyptus (OLE), para-menthane-diol (PMD), or 2-undecanone. Wearing long pants, long sleeves, and high socks helps protect the skin from insect bites. Any attached ticks should be promptly removed using fine-tipped tweezers. Drinking untreated surface water should also be avoided. During mowing or landscaping, it is essential not to mow over sick or dead animals. Checking the area beforehand can help reduce this risk. Although not yet formally studied, wearing masks while mowing may lower the risk of inhaling bacteria. For those who hunt, trap, or handle animals, particularly rabbits, muskrats, prairie dogs, and other rodents, wearing gloves is recommended. Game meat should always be cooked thoroughly before consumption [183].

In the 1960s, a live vaccine strain (LVS) *Francisella* vaccine was developed in the Soviet Union, attenuating *F. holarctica* [184]. This vaccine is currently not licensed in the US due to its inability to provide complete protection against virulent human *F. tularensis.* It concerns that it may revert to a more virulent form of the bacterium [185]. Although protection is dose-dependent and route-dependent, this demonstrates that the vaccine is not sufficient to protect against virulent *Francisella* exposure in most cases fully [185].

### 6.6. Recommendations

In endemic areas, individuals should protect themselves by using insect repellents containing DEET, picaridin, IR3535, oil of lemon eucalyptus (OLE), para-menthane-diol (PMD), or 2-undecanone. When entering wildlife areas, it is essential to wear light-colored, long clothing that fully covers the legs and arms to reduce exposure to ticks and other vectors. After engaging in outdoor activities such as hiking, camping, or trekking, individuals should carefully inspect their bodies for ticks. Gardeners should wear appropriate personal protective equipment (PPE), and all individuals should avoid contact with animal carcasses. Garbage should be collected regularly, and food scraps should not be left exposed; food must be stored in safe places. Unfrequented areas should be cleaned routinely to prevent the accumulation of vegetation and debris that could provide shelter for animals such as rodents. Hunters, especially in endemic areas, must use PPE such as rubber gloves and face masks when handling rabbits, hares, and rodents, and should wash their hands thoroughly with soap and water afterward. Wild animal and game meat must be thoroughly cooked before consumption. Additionally, people should avoid drinking untreated water from lakes and rivers and must ensure vegetables are properly washed before eating.

## 7. *Borrelia*

Lyme disease is a bacterial illness transmitted to humans through the bite of infected ticks. The disease is caused by bacteria in the family Borreliaceae, particularly *Borrelia (Borreliella) burgdorferi* s.l. Ticks become infected by feeding on animals that carry the bacteria in their blood. The bacteria are only transmitted by the bites of the ticks *Ixodes ricinus* and *I. persulcatus* (the former is common in most of Europe, while the latter is found in the Baltic countries and Finland). In the most affected regions, tick infection rates may exceed 10%. These areas are mainly located in central Europe; however, in recent years, there has been a spread of infected ticks toward northern latitudes (i.e., Scandinavia) [186].

### 7.1. Etiology

Lyme borreliosis (LB) is the most common tick-borne disease in the Northern Hemisphere [187,188]. It is a zoonotic disease caused by the Gram-negative spirochete bacteria belonging to the family Spirochaetaceae, specifically the *Borrelia burgdorferi* sensu lato complex (*B. burgdorferi* s.l.). The complex is divided into genospecies, including *B. afzelii*, *B. burgdorferi* sensu stricto, *B. garinii*, and possibly *B. valaisiana* [189,190,191].

### 7.2. Epidemiology

Approximately 476,000 cases are reported annually in the United States due to *B. burgdorferi* [192], and around 200,000 in Europe [187]. The disease is transmitted by *Ixodes* ticks, with rodents being the primary hosts of the bacteria [193]. In Europe, *Ixodes ricinus* is the primary vector of *B. burgdorferi* s.l. [194]. In eastern and central North America, the black-legged tick *Ixodes scapularis* is the primary vector [195,196]. Other species, such as *Ixodes persulcatus* and *Ixodes pacificus*, also contribute to transmission [197]. Among the reservoirs, the white-footed mouse (*Peromyscus leucopus*), native to eastern North America [198]; it is highly susceptible to infection [199,200].

**Figure 6:**
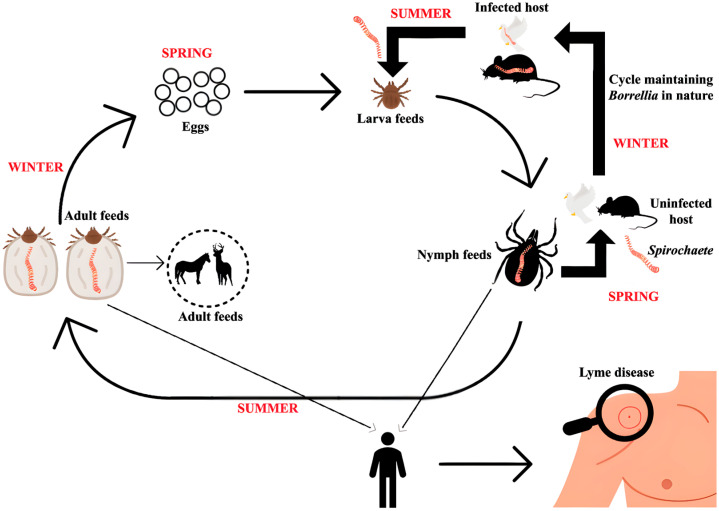
Transmission cycle of *Borrelia* species. Rodents act as reservoir hosts, maintaining *Borrelia* in nature and supporting its persistence. Larval and nymphal ticks acquire the bacteria when feeding on infected rodents. Infected nymphs and adult ticks can then transmit *Borrelia* to vertebrate hosts, including humans, during blood meals. This cycle, involving rodents and ticks at different life stages, facilitates the continued circulation and environmental spread of *Borrelia*.

Ticks carrying pathogenic strains of *B. burgdorferi* transmit infection while feeding on vertebrate hosts, such as humans [201,202,203]. Transmission occurs 48 to 72 h after the tick first attaches to the host [202]. Infected ticks are unlikely to transmit the organisms within the first few hours after feeding; however, the risk increases gradually with the duration of the blood meal [204] (Figure 6).

The enzootic cycle of Lyme borreliosis involves *Ixodes* ticks transitioning through larval, nymph, and adult stages. During their larval stage, ticks become infected with *Borrelia burgdorferi* sensu lato by feeding on infected reservoir hosts, primarily small mammals. Migratory birds play a dual role by spreading the spirochetes geographically and acting as reservoir hosts themselves. Humans, however, are incidental hosts who do not contribute to the transmission cycle.

Notably, climate change is expected to accelerate the spread of LB, making it crucial to implement preventive measures, including epidemiological and molecular surveillance, as well as promoting health education to the general public [205].

### 7.3. Clinical Signs

In humans, the disease has an incubation period of 3 to 30 days [206]. LB can manifest clinical signs such as fatigue, hyperemia, myalgia, erythema migrans, and cardiac and neurological signs [207]. In Europe, neuroborreliosis is the most frequently observed neurological sign in the early phase of LB [208].

In animals, clinical signs are barely noticeable [209,210]. In white-footed mice (*Peromyscus leucopus*), no effect of the infection on animal survival was observed [210,211].

In dogs, lameness may be observed, often with associated hyperemia and anorexia. Arthritis commonly occurs in a single joint, most often in the carpus or tarsus [212]. Clinical signs observed in horses include lethargy, low-grade fever, and stiffness and swelling of the distal appendicular joints [213].

### 7.4. Diagnosis

According to CDC guidelines, a two-step testing process should be adopted for serological testing for Lyme disease. Both steps are required and can be done using the same blood sample. If this first step is negative, no further testing is necessary. However, if the first step yields a positive or indeterminate result (sometimes referred to as “equivocal”), the second step should be performed. The final result is only positive when the first test is positive (or equivocal) and the second test is positive (or, for some tests, equivocal). Standard two-tier testing (STTT) employs enzyme immunoassay (EIA) as the initial step and Western blotting (WB) as the second step. Increasingly, laboratories are using modified two-tier testing (MTTT) in which both assays are EIAs [214].

### 7.5. Treatment, Prevention, and Control

Prevention of Lyme borreliosis following a tick bite has been reported using a single dose of doxycycline [215]. The use of doxycycline, amoxicillin, and cefuroxime axetil for 14 days in patients with advanced clinical symptoms has also demonstrated efficacy [216].

The primary methods of preventing infection are avoiding tick bites and promptly removing attached ticks. The most effective tick-bite avoidance strategies include wearing protective clothing, like long trousers and long-sleeved shirts, and using tick repellents. The skin should be checked periodically for attached ticks, which should be removed using tweezers or fine-pointed forceps. For safe removal, grasp the tick as closely as possible to the skin, pulling gently upwards and trying not to break off the mouthparts. The risk of borrelial infection is not increased if the tick’s mouth parts are left behind. It is recommended to use a skin disinfectant after tick removal to prevent pyogenic infection. When searching for attached ticks, pay particular attention to skin folds, the groin area, armpits, under the breasts, the waistband area, and the backs of the knees, as ticks tend to seek out more humid areas for attachment. In children, the head, including the scalp, and neck should be carefully checked, as tick bites are relatively more common in these areas in this age group. An effective preventive measure for individuals with intense tick exposure, such as forestry workers, rural workers, or military personnel on active duty, is the use of permethrin-impregnated clothing [204].

Targeting ticks on mice with acaricides has also been used, either by using bait boxes that coat mice as they enter a feeder, nesting material impregnated with an acaricide that transfers the agent to the fur of mice in the nest, or oral feeding with baits containing acaricides [217].

Currently, there is no vaccine available for humans against Lyme disease, also known as Lyme borreliosis [218]. However, an alternative vaccine is the OspA-based vaccine (LYMErix). Outer surface protein A (OspA) has been the basis for at least two different vaccines, LYMErix (SmithKline Beecham) and ImuLyme (Pasteur-Mé-rieux-Connaught). However, only LYMErix was licensed and available to consumers from 1998 until 2002, when it was voluntarily withdrawn from the market [219].

### 7.6. Recommendations

To prevent exposure to rodents and ticks, it is essential to implement preventive rodent control measures, such as avoiding the accumulation of garbage and debris, storing food in tightly sealed containers, and installing metal screens on air and sewage inlets. People should avoid areas where ticks may be present, such as lawns, forests, and wildlife habitats. The use of effective tick repellents is highly recommended. In endemic regions, individuals should apply insect repellents containing DEET, picaridin, IR3535, oil of lemon eucalyptus (OLE), para-menthane-diol (PMD), or 2-undecanone, and wear light-colored, long clothing that fully covers the legs and arms when entering wildlife areas. After potential exposure to ticks, such as visiting tick-infested environments, individuals should promptly remove their clothing, wash it, take a shower, and carefully inspect their body, especially hairy areas, for ticks. If a tick is found, it should be removed using fine-tipped tweezers, gripping it at the head or mouth parts close to the skin and pulling it out slowly. Ticks should never be handled with bare or unprotected hands. When hiking or trekking, it is also advised to avoid sitting on the ground, vegetation, or rocks, and to stay on designated paths and trails to minimize contact with ticks.

## 8. *Rickettsia*

Rickettsiae are intracellular bacteria responsible for causing vector-borne zoonotic diseases worldwide [220,221]. *Rickettsiae* can be transmitted by ticks, fleas, lice, and mites, infecting domestic animals, wild animals, and humans, thus representing diseases of direct implication to human health [222].

### 8.1. Etiology

Rickettsioses (Rickettsiales: Rickettsiaceae) are diseases that severely impact public health, caused by intracellular, Gram-negative bacteria transmitted by ticks, performing enzootic or epizootic cycles in wild vertebrate hosts [223]. These diseases have gained greater notoriety in the medical and scientific communities in recent years [224]. In recent years, several species of bacteria have been incriminated as pathogens in humans [220]. This bacterial genus can be divided into the spotted fever group (SFG) and the typhus group (TG) [225], with the majority belonging to the central group of classical spotted fever [226].

### 8.2. Epidemiology

Regardless of the length of travel (short- or long-term), all age groups are at risk for rickettsial infections during visits to endemic areas. Transmission risk increases with the time spent participating in outdoor activities, particularly during seasons when the vector is at its peak in terms of feeding and lifecycle activity. In many parts of the world, however, rickettsial infections occur year-round. The most commonly diagnosed rickettsial diseases in travelers belong to the spotted fever or typhus groups; notably, rickettsial infections can also be caused by emerging and newly recognized species [227]. Numerous species of *Rickettsia* are associated with diseases in humans, as well as various vectors (Table 1).

**Figure 7:**
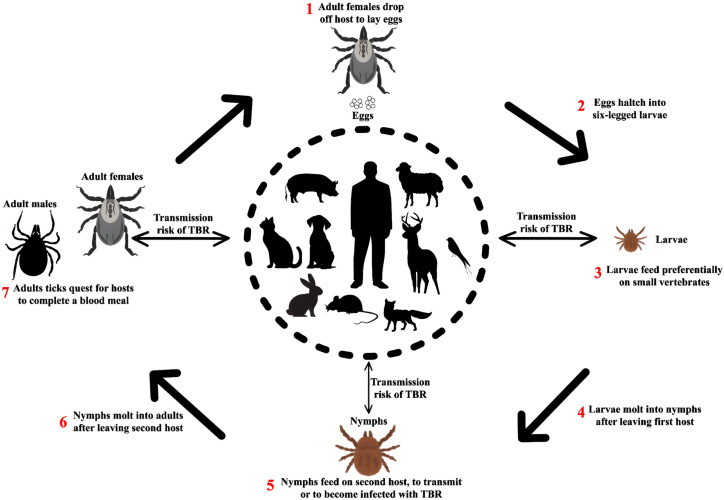
Transmission of *Rickettsia* to humans by ticks and its natural life cycle. Rodents act as reservoir hosts, perpetuating *Rickettsia* in nature and maintaining the epidemiological cycle. Larval and nymphal ticks acquire the bacteria when feeding on infected rodents. Infected nymphs and adult ticks can then transmit *Rickettsia* to vertebrate hosts, including humans, during blood meals.

Most rickettsial pathogens are transmitted directly to humans by infected arthropod vectors (i.e., fleas, lice, mites, or ticks) during feeding. In addition, pathogens can also be transmitted when a person accidentally inoculates Rickettsiae into the arthropod bite wound (or other breaks in the skin); this transmission can occur by scratching skin contaminated with infectious arthropod fluids or feces, or by crushing the arthropod vector at the bite site. Another possible transmission route is through inhalation of Rickettsiae or inoculation of the conjunctiva with infectious material. While possible, transmission of some rickettsiae through transfusion of infected blood products or organ transplants is less common, especially *Anaplasma* and *Ehrlichia* species [227] (Figure 7).

The impact of climate change on vector-borne and rodent-borne infectious diseases is essential [167]. The increase in rickettsial infections is catalyzed by several factors, including climate change [220,238,239].

### 8.3. Clinical Signs

Several *Rickettsia* species are responsible for distinct illnesses within the spotted fever and typhus group, each with characteristic clinical presentations. *R. conorii* causes Mediterranean spotted fever (MSF), marked by fever, headache, a maculopapular rash affecting the palms and soles, and a black eschar (“tache noire”) at the tick bite site [240]. *R. rickettsii* leads to Rocky Mountain spotted fever (RMSF), presenting with fever, headache, muscle pain, and a maculopapular rash that may become purpuric in severe cases [231]. Japanese spotted fever (JSF), caused by *Rickettsia japonica*, typically presents with symptoms including fever, asthenia, myalgia, rash, and anorexia [234]. *R. africae* is responsible for African tick-bite fever (ATBF), characterized by fever, skin lesions (eschar), rash, lymphangitis, headache, myalgia, and lymphadenopathy [241]. *R. conorii* can develop Mediterranean spotted fever (MSF), resulting in clinical symptoms such as fever, headache, maculopapular rash affecting the palms of the hands and soles of the feet, and the presence of an eschar, the “tache noire”, at the site of the tick bite [240].

In a study evaluating clinically ill cats for evidence of rickettsial infection, no association was found between antibody positivity and fever, and no febrile cats tested positive for *R. felis* or *R. rickettsii* [242]. In an experimental infection of dogs with a Brazilian strain of *Rickettsia rickettsi* fever, it was possible to observe lethargy, anorexia, ocular lesions, thrombocytopenia, and anemia [243]. In dogs, RMSF manifests with fever, lethargy, decreased appetite, tremors, scleral injection, a maculopapular rash on the ears and exposed skin, and petechial lesions on the mucous membranes [243,244,245].

### 8.4. Diagnosis

Methods for diagnosing rickettsial infections include serology, such as indirect immunofluorescence assay and enzyme-linked immunosorbent assay (ELISA) for IgM or IgG [246]. Molecular tests, such as PCR, are capable of detecting rickettsiae in the acute phase of the disease [247].

### 8.5. Treatment, Prevention, and Control

In infections caused by *Rickettsia conorii*, the gold standard treatment is doxycycline at a dose of 200 mg per day [240]. Doxycycline is also the treatment of choice in cases of infection by *Rickettsia rickettsii* [248], *Rickettsia japonica* [249], *Rickettsia typhi* [250], *Rickettsia prowazekii* [251], *Rickettsia akari* [248], and *Rickettsia africae* [252].

There is no vaccine available to prevent rickettsial infections. Antibiotic prophylaxis is not recommended for rickettsiae, and antimicrobial agents should not be given to asymptomatic individuals. Travelers to areas endemic for rickettsiae should seek to minimize their exposure to infectious arthropods (including fleas, lice, mites, and ticks) and avoid animal reservoirs (particularly dogs and rats) [227].

It is not necessary to exclude individuals with rickettsial infections from daycare, preschool, school, or the workplace. It is recommended that long-sleeved protective clothing and a wide-brimmed hat be worn to reduce the risk of infection when engaging in activities where human contact with ticks, lice, mites, or fleas may occur, such as hiking and camping in areas where these pests are present. It is also recommended that you use insect repellent containing DEET or picaridin and examine your skin for possible bites (especially behind the ears, on the back of the head, in the groin, armpits, and behind the knees) [253].

The first whole-cell antigen (WCA) vaccines against *R. prowazekii* and *R. rickettsii* were produced in the 1920s. This vaccine was used on German soldiers during World War II [254]. A similar vaccine was developed by the US military, which helped alleviate the disease [255]. Another vaccine option against epidemic typhus was produced by isolating *R. prowazekii* from the lungs of infected rabbits (Castaneda vaccine) [255] or from the vaginal tunica and peritoneum of infected rats (Zinsser-Castaneda vaccine) [256]. Similarly, in the 1970s, formalin-inactivated *R. rickettsii* in chicken embryonic fibroblasts [257,258] protected monkeys [259,260]. This vaccine ameliorated the disease in humans but did not prevent infection [261]. One alternative is the use of avirulent or attenuated bacteria, such as low-virulence strains of *O. tsutsugamushi*, which effectively induce the human immune system [262]. New strategies and vaccines with good immune induction, prolonged protection, and large-scale production capacity are needed.

### 8.6. Recommendations

In endemic areas, integrated pest control is crucial for disease prevention. It should involve a thorough environmental assessment, the use of physical barriers, controlled chemical applications, continuous monitoring, and community-based environmental education. Keeping pets dewormed is an essential preventive measure. Individuals in these regions should use insect repellents containing DEET, picaridin, IR3535, oil of lemon eucalyptus (OLE), para-menthane-diol (PMD), or 2-undecanone, especially when entering areas with wildlife. It is also advisable to wear light-colored, long-sleeved clothing that covers the legs and arms. During outdoor activities such as hiking or camping, individuals should stay on safe trails and avoid contact with potentially infested environments. Hunters, particularly in endemic regions, must use personal protective equipment (PPE), such as rubber gloves and face masks, when handling animals like rabbits, hares, and rodents. They should then wash their hands thoroughly with soap and water to minimize the risk of infection.

## 9. Future Research

**Figure 8:**
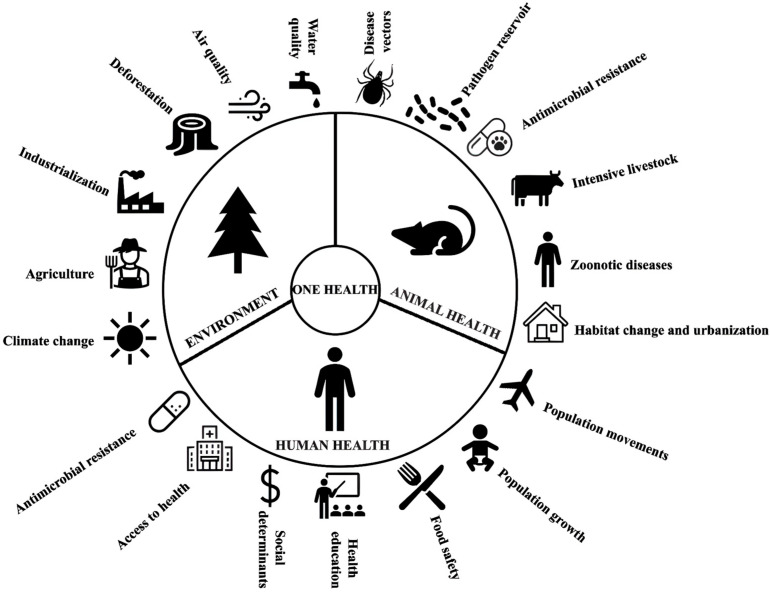
The role of rodents in the One Health context: integrating environmental, animal, and human health to understand and prevent rodent-borne diseases.

We must understand that rodent-borne diseases are fully embedded in the context of One Health (Figure 8). Therefore, broader and more in-depth research is needed on the epidemiological role of rodents as pathogen reservoirs and their relationship within the One Health context [4]. It is crucial to study how deforestation, urbanization, and climate change can alter rodent behavior and distribution, influencing the risk of pathogen transmission [263]. At the same time, rodent genomic and microbiota analysis must be expanded to identify genes associated with resistance or susceptibility to pathogens. The use of artificial intelligence must be explored and utilized rationally and positively, such as in the development of mathematical and computational models that connect ecological, climatic, and population information and data to predict outbreaks of rodent-borne diseases. At the same time, we must seek innovative and sustainable methods of rodent control.

## 10. General Recommendations

To efficiently manage rodent-borne zoonotic illnesses, the following priorities are recommended:

1. Public Health and Prevention
  − Awareness Campaigns: Educate communities endemic for rodents and rodent-borne diseases on rodent-borne illnesses, transmission, and preventive measures.
  − Personal Protection: Promote the utilization of protective equipment, insect repellents (e.g., DEET, picaridin), and proper hygiene measures.
  − Rodent Control: Employ integrated pest management (IPM) practices, such as sanitation, rodent-proofing buildings, and secure waste disposal.
  − Vector Control: Regulate tick and flea populations through environmental management, acaricides, and treatment of pets.
2. Health care and Diagnostics
  − Early Diagnosis: Enhance laboratory infrastructure to achieve early diagnosis of zoonotic pathogens using molecular (PCR) and serological diagnostic methods.
  − Antibiotic Stewardship: Support proper use of antimicrobials to prevent resistance, particularly in leptospirosis and rat-bite fever.
  − Vaccination Research: Develop vaccine research for high-mortality diseases like plague and tularemia.
3. Environmental and One Health Strategies
  − Climate Adaptation: Monitor climate-driven fluctuations in rodent and vector populations for predicting disease outbreaks.
  − Ecosystem Management: Re-establish ecosystems to minimize human-rodent interaction and maintain biodiversity.
  − Wildlife Surveillance: Regularly monitor rodent and arthropod populations in high-risk zones.
4. Policy and Global Coordination
  − International Reporting: Improve international surveillance networks for prompt reporting of zoonotic outbreaks.
  − Regulatory Measures: Enact stricter rodent trade, pet keeping, and food hygiene regulations to minimize risks of transmission.
  − One Health Frameworks: Promote interspecies collaboration among veterinarians, ecologists, and public health professionals to respond to zoonotic threats integratively.

## 11. Conclusions

Rodents play a significant but often underrecognized role in the emergence and transmission of zoonotic bacterial infections. Due to their ability to adapt to diverse habitats, including cities, they coexist closely with human and domestic animal populations. This review highlights the major public health threats posed by rodent-borne pathogens, including *Leptospira*, *Streptobacillus moniliformis*, *Yersinia pestis*, *Salmonella*, *Francisella tularensis*, *Borrelia burgdorferi*, and *Rickettsia* spp. These agents cause diseases that range across the spectrum from mild febrile illnesses to life-threatening illnesses, with transmission occurring through direct contact, a contaminated environment, or an arthropod vector. Climate change, urbanization, and habitat encroachment facilitate the transmission of these diseases by altering rodent population dynamics, vector distribution, and increasing the risk of exposure to humans. The One Health approach, which synthesizes human-animal-environment interactions, is essential in managing these risks. Increased surveillance, diagnostic capabilities, and interdisciplinary coordination are the building blocks for preventing outbreaks and reducing disease burden. Rodents are sneaky carriers of deadly pathogens that necessitate concerted and preventive measures to stop their impact. Through a synergistic approach combining scientific research, public health interventions, and environmental conservation, it is possible to reduce the prevalence of rodent-borne diseases and safeguard global health in an era of environmental and climatic change.

## Figures and Tables

**Table 1:** Main features of rickettsiosis.

Transmission	Form of the Disease	Etiology	Vector	Animal Host(s)	Reference
Lice	Epidemic typhus	*Rickettsia prowazekii*	*Pediculus humanus corporis*	Humans flying squirrels	[228]
	Endemic typhus or murine typhus	*Rickettsia typhi*	*X. cheopis*	Rodents	[229]
Ticks	Tick-Borne Lymphadenopathy, Dermacentor-Borne-Necrosis-Erythema-Lymphadenopathy, Scalp Eschar Neck Lymphadenopathy, Mediterranean spotted fever (MSF)	*Rickettsia slovaca*, *Candidatus Rickettsia rioja*, *Rickettsia raoultii*, *Rickettsia conorii*	*Dermacentor marginatus*, *Dermacentor reticulatus*, *Rhipicephalus sanguineus*	Dogs, rodents	[230]
	Rocky Mountain spotted fever (RMSF), Brazilian spotted fever (BSF)	*Rickettsia rickettsii*	*Dermacentor* spp., *Rhipicephalus sanguineus*, *Amblyoma* spp.	Rodents	[231,232]
	Japanese spotted fever (JSF)	*Rickettsia japonica*	*Dermacentor taiwanensis*,	Rodents	[233,234]
			*Haemaphysalis flava*, *Ixodes* spp.		
	African tick-bite fever (ATBF)	*Rickettsia africae*	*Amblyomma* spp., *Rhipicephalus* spp., *Hyalomma*	Domestic and wild ruminants	[235]
Mites	Smallpox (rickettsialpox)	*Rickettsia akari*	*Liponyssoides sanguineus*	House mice, wild rodents	[236,237]

## Data Availability

No new data were created or analyzed in this study.
